# Changes in Nutritional Status and Bioimpedance Parameters During EAA/KA Supplementation in Elderly Patients Undergoing Maintenance Hemodialysis: A Retrospective Observational Study

**DOI:** 10.3390/nu18213620

**Published:** 2026-10-31

**Authors:** Sandra Papalini, Arianna Ceravolo, Eleonora Albanese, Fatma Cossetti, Laura Pettorini, Emma Espinosa

**Affiliations:** 1 Nephrology and Dialysis Unit, ASL ROMA1 PO S. Spirito, 00193 Rome, Italy; 2 Bachelor of Science in Nursing, UNIROMA1 Sapienza University, 00185 Rome, Italy; 3 Clinical and Molecular Sciences Department, Università Politecnica delle Marche, 60126 Ancona, Italy; e.espinosa@univpm.it; 4 Geriatrics Unit, Santa Croce Hospital, 61032 Fano, Italy

**Keywords:** protein-energy wasting (PEW), malnutrition, hemodialysis (HD), essential amino acids and keto-analogues (EAA/KA), chronic kidney disease (CKD)

## Abstract

**Background/Objectives**: Protein Energy Wasting (PEW) is common among patients undergoing maintenance hemodialysis (HD), particularly older adults, and is associated with increased frailty, susceptibility to infections, hospitalization, and mortality. Nutritional counseling alone may be insufficient to improve nutritional status in some patients. Supplementation with essential amino acids and ketoanalogues (EAA/KA) may support nutritional status in patients undergoing HD. This study aimed to evaluate changes in nutritional and bioimpedance parameters in older patients undergoing maintenance HD and receiving EAA/KA supplementation. **Methods:** This retrospective observational study evaluated changes over six months in 10 patients undergoing maintenance HD with a baseline Malnutrition–Inflammation Score (MIS) greater than 7 who received EAA/KA supplementation. **Results:** After six months, serum albumin levels increased significantly (*p* < 0.001), while extracellular water (ECW) decreased (*p* = 0.024). Bioelectrical impedance analysis (BIA) also showed a significant increase in phase angle (PA; *p* = 0.029) and significant decreases in the extracellular mass-to-body cell mass ratio (ECM/BCM; *p* = 0.034) and the sodium-to-potassium ratio (Na/K; *p* = 0.025). The MIS also decreased significantly (*p* = 0.003). **Conclusions:** In this small retrospective study, six months of EAA/KA supplementation were associated with improvements in serum albumin, MIS, and several bioimpedance parameters in patients undergoing maintenance HD. These preliminary findings suggest a potential role for EAA/KA supplementation in the nutritional management of patients at nutritional risk. However, the small sample size, retrospective design, and absence of a control group limit the interpretation of these findings. Further prospective controlled studies involving larger populations are needed to confirm these observations.

## 1. Introduction

PEW is characterized by a reduction in body protein and energy stores and is often associated with impaired functional capacity in patients with end-stage kidney disease [1,2]. In patients with chronic kidney disease (CKD), particularly those undergoing HD, PEW is associated with adverse clinical outcomes, increased hospitalization rates, and higher mortality [3].

A meta-analysis by Carrero et al. [4] reported a prevalence of PEW ranging from 28% to 54% among patients receiving maintenance HD.

The pathogenesis of PEW in CKD is multifactorial and closely related to the metabolic disturbances associated with uremia [5]. Chronic inflammation, metabolic abnormalities, and metabolic acidosis may promote protein catabolism and increase energy expenditure. These processes, together with reduced dietary intake, protein losses during dialysis, and physical inactivity, contribute to the development and persistence of PEW [6,7,8,9].

PEW is associated with increased morbidity and mortality, loss of muscle mass, frailty, and impaired quality of life. Early identification and appropriate nutritional management are therefore essential. The KDIGO 2024 Clinical Practice Guideline emphasizes the role of renal dietitians and qualified nutrition professionals in providing individualized dietary counselling for patients with CKD [10].

According to the KDOQI 2020 Clinical Practice Guidelines, metabolically stable adults undergoing maintenance HD should generally receive a daily protein intake of 1.0–1.2 g/kg body weight and an energy intake of 25–35 kcal/kg body weight, individualized according to age, physical activity, body composition, clinical condition, and nutritional goals [11].

MIS [12] is a validated, simple, and cost-effective tool for assessing nutritional and inflammatory status in HD patients. It comprises 10 components covering medical history, dietary intake, anthropometric measurements, laboratory parameters, and functional capacity. Several studies have investigated the associations between MIS and quality of life, comorbidities, and mortality in patients with CKD [13,14,15,16]. In particular, an MIS score above 7 has been associated with an increased risk of mortality [13]. Based on this evidence, we adopted an MIS cut-off of >7 to identify patients at high nutritional risk.

EAA/KA formulations are primarily used to support nutritional management in selected patients with advanced CKD following low- or very-low-protein diets [17,18,19]. Ketoanalogues, commonly administered as calcium salts, provide nitrogen-free precursors of essential amino acids, while essential amino acid supplementation supports protein synthesis. Following absorption, ketoanalogues can undergo transamination by accepting amino groups from non-essential amino acids, thereby facilitating nitrogen reutilization for amino acid synthesis and potentially limiting nitrogenous waste production.

Although EAA/KA supplementation is traditionally associated with protein-restricted diets in patients with advanced CKD who are not yet receiving dialysis, this approach may also have a role in selected HD patients with PEW. Studies investigating EAA/KA supplementation in HD patients [20,21,22,23,24,25] have reported improvements in serum albumin levels, Subjective Global Assessment (SGA) scores, and appetite. Potential benefits have also been described in anemia management [26] and in calcium–phosphorus metabolism and parathyroid hormone (PTH) regulation [27,28]. A reduction in peripheral edema has also been reported [23].

Given the clinical burden of PEW in HD patients and the limited evidence regarding the nutritional effects of EAA/KA supplementation in this population, the present study aimed to evaluate changes in nutritional status in elderly patients undergoing maintenance HD and treated with EAA/KA over a six-month observation period.

## 2. Materials and Methods

**Study design and population.** This retrospective observational study evaluated patients undergoing maintenance HD at our dialysis center who had a MIS > 7 at baseline [13]. Based on their nutritional status, EAA/KA supplementation was prescribed. No exclusion criteria were applied. Fourteen patients initiated EAA/KA supplementation during the study period. Of these, 10 completed the six-month follow-up and had measurements available at both baseline (T0) and six months (T6); these 10 patients constituted the population included in the primary paired analysis.

The resulting study population was predominantly elderly and characterized by a substantial burden of comorbidities. As shown in Table 1, the high Charlson Comorbidity Index (CCI) [29] scores reflect the considerable clinical complexity of the study population.

According to the KDOQI 2020 Clinical Practice Guideline, patients were prescribed a diet providing 1.2 g of protein/kg body weight/day and 30 kcal/kg body weight/day.

Nutritional status was assessed at baseline and after 3 and 6 months, in accordance with the nutritional monitoring protocol established by the Lazio Regional Health Authority for patients undergoing maintenance HD.

During the observation period, patients also received erythropoietin alfa, iron supplementation with ferric carboxymaltose (FCM), a vitamin D analogue (paricalcitol), a calcimimetic agent (etelcalcetide), a phosphate binder (sucroferric oxyhydroxide, SFO), and statin therapy (simvastatin). The medications and their respective dosages at baseline and at the end of the observation period are reported in Table 2.

Based on previously published studies [20,21,22,23,24,25,26,27,28], EAA/KA supplementation was prescribed at a dose of one tablet per 10 kg of body weight, corresponding to 60 mg/kg body weight. This dose was lower than that commonly used in patients with CKD receiving conservative treatment and following low- or very-low-protein diets, owing to the calcium content of the tablets (Appendix A, Table A3).

The EAA/KA supplements were dispensed directly by the dialysis center upon patients’ request, and each dispensing was recorded. Treatment adherence was assessed by pill count.

**Nutritional Assessment and Data Collection.** The nutritional assessments included in this study were those prescribed by the Lazio Regional Health Authority for monitoring the nutritional status of patients undergoing maintenance HD. Assessments were performed every three months.

**Blood sampling and dialysis adequacy.** Blood samples were collected before the first dialysis session following the longest interdialytic interval. The timing of blood sampling followed standard clinical practice and is described in Appendix A. Laboratory assessments included hemoglobin, complete blood count, iron metabolism parameters (serum iron, transferrin, and ferritin), and serum creatinine. Pre- and post-dialysis blood urea levels were measured to calculate single-pool Kt/V (spKt/V), an index of dialysis adequacy, and the normalized protein catabolic rate (nPCR), an indirect indicator of dietary protein intake, using Daugirdas’ single-pool equations.

**Nutritional and biochemical parameters.** Nutritional indicators included serum albumin, measured by protein electrophoresis, transferrin, total cholesterol, and triglyceride levels.

**Mineral metabolism.** Serum calcium, phosphorus, and parathyroid hormone (PTH) levels were measured to assess mineral metabolism.

**Anthropometric measurements.** Body mass index (BMI) was calculated using post-dialysis body weight measured at the midweek hemodialysis session.

**Bioelectrical impedance analysis.** Body composition was assessed using a BIA 101 device (Akern^®^, RJL Systems, Florence, Italy). Measurements were performed 20 min after the end of the midweek hemodialysis session. The timing of BIA measurements followed standard clinical practice and is described in Appendix A. Impedance data were processed using Akern^®^ software Bodygram PLUS 1.2.2 and Jamovi 2.6.44. In addition to standard impedance-derived parameters, extracellular mass-to-body cell mass ratio (ECM/BCM) was evaluated. Previous studies have reported that ECM/BCM values greater than 1.2 in HD patients are associated with malnutrition and correlate significantly with MIS and mortality [30,31,32,33,34].

**Malnutrition–Inflammation Score.** The MIS was calculated according to the method described in the literature [12]. The criteria used for score calculation are presented in Appendix A (Table A1).

**Statistical analysis:** Statistical analyses were performed using Jamovi 2.6.44 software [35]. Descriptive statistics are used to summarize the study variables. Nutritional assessments were performed at baseline (T0), at three months (T3), and at six months (T6) in all 10 patients who completed the study. The primary analysis focused on within-patient changes in continuous variables between baseline and the end of the six-month observation period. These changes were assessed using the paired Student’s *t*-test. The normality of the within-subject differences (T6–T0) was assessed using Q–Q plots and the Shapiro–Wilk test. Mean differences (T6–T0) and their corresponding 95% confidence intervals (CIs) were calculated to quantify the magnitude and precision of the observed changes and to provide preliminary estimates to inform sample size calculations for future studies [36]. The longitudinal evolution of selected parameters across T0, T3, and T6 is presented graphically using box plots. Changes in individual MIS components between baseline and six months were explored using the Wilcoxon signed-rank test for paired observations, given the ordinal nature of the item scores and the small sample size. All tests were two-sided, and *p* values < 0.05 were considered statistically significant.

## 3. Results

The observation period extended from March 2024 to June 2026. During this period, 14 patients initiated treatment, of whom 10 completed the 6-month follow-up. Four patients discontinued the study: two were hospitalized, one due to cognitive decline and the other due to metastatic cancer; one patient died from an unspecified cause while travelling; and one patient was excluded because of poor treatment adherence.

All patients underwent bicarbonate hemodialysis three times per week and were anuric, with no residual renal function (RRF).

Baseline patient characteristics are presented in Table 1. EAA/KA supplementation was prescribed on the basis of nutritional status, defined as an MIS score > 7, irrespective of dialysis vintage or the presence of severe comorbidities, including cognitive impairment (n = 2) and cancer (n = 3). CCI was also calculated to assess the overall comorbidity burden [29]. All patients had a history of hypertension. However, the primary etiology of kidney failure could not be definitively established, as no patient had undergone a kidney biopsy.

**Table 1:** Baseline patient characteristics.

	N (%)	Median (IQR)
Female	4 (40)	
Age (yrs)		82.5 (6.5)
Duration of dialysis (months)		10 (9.5)
CCI		8.5 (1.75)
Comorbidities *		
Hypertension	10 (100)	
Multiple myeloma	1 (10)	
Lung cancer	1 (10)	
Intestinal cancer	1 (10)	
Severe vascular disease	2 (20)	
Type 1 diabetes mellitus	1 (10)	
Cognitive impairment	2 (20)	

* Comorbidities are not mutually exclusive.

Table 2 summarizes the pharmacological treatments and their respective dosages at baseline and at the end of the 6-month follow-up.

**Table 2:** Pharmacological Therapy.

	T0	T6
EPO-α (IU/week)	14,240 ± 12,819	8100 ± 5173
FCM (mg/week)	40 ± 52	10 ± 32
Paricalcitol (mcg/week)	5.6 ± 4.2	5.6 ± 2.6
Etecalcetide (mg/week)	3.75 ± 6.2	3.5 ± 4.3
SFO (mg/die)	305 ± 463	520 ± 374
Simvastatin (mg/die)	11 ± 25	11 ± 25

Table 3 summarizes the nutritional, anthropometric, biochemical, dialysis-related, and body composition parameters measured at baseline (T0) and after 6 months of treatment with EAA/KA (T6).

Due to the small sample size, mean differences between baseline (T0) and the 6-month follow-up (T6) were also calculated to quantify the magnitude of the observed changes, as reported in Table 4.

The main findings were an increase in serum albumin levels (*p* < 0.001; mean difference, +0.39 g/dL) and a reduction in MIS (*p* = 0.003; mean difference, −5.50). Both changes may be clinically relevant. Significant reductions were also observed in the ECM/BCM (*p* = 0.034; mean difference, −0.31) and extracellular water expressed as a percentage of total body water (ECW%; *p* = 0.025). In contrast, body weight and BMI remained substantially unchanged. Bold type indicates statistically significant confidence intervals.

Regarding biochemical parameters, serum calcium levels increased significantly (*p* = 0.024), while remaining within the normal range. Serum total cholesterol levels also increased, although the change did not reach statistical significance (*p* = 0.084). The corresponding mean differences were small (+0.37 mg/dL for serum calcium and +7 mg/dL for total cholesterol). No significant changes were observed in complete blood count parameters. Mean serum phosphorus levels increased, whereas PTH levels decreased; neither change was statistically significant.

Dialysis-related parameters, including predialysis serum urea levels, spKt/V, and nPCR, showed no statistically significant changes over the observation period.

BIA showed a significant increase in phase angle (PA; *p* = 0.029), together with significant decreases in ECM/BCM (*p* = 0.034) and the Na/K (*p* = 0.025). These findings, alongside the reduction in extracellular water (ECW) in absolute terms (*p* = 0.024) and as a percentage of total body water (ECW%; *p* = 0.025), are consistent with an improvement in hydration status and may indicate a favorable change in body composition. Increases in body cell mass (BCM; *p* = 0.070) and body cell mass index (BCMI; *p* = 0.078) were also observed, although these changes did not reach statistical significance. No statistically significant changes were observed in muscle mass (MM) or appendicular skeletal muscle mass (ASMM).

The box plots in Figure 1 illustrate the three-monthly trends in serum albumin, MIS, BMI, BCMI, ECW%, and the ECM/BCM ratio over the six-month observation period.

## 4. Discussion

The main limitation of this study is the small sample size. The study population consisted of elderly patients undergoing maintenance HD, with a substantial burden of comorbidities. Hospitalizations, clinical complications, and difficulties with treatment adherence made it challenging to obtain complete six-month follow-up data. Indeed, only 10 of the 14 patients who initiated EAA/KA supplementation completed the observation period.

Another important limitation is the absence of a control group. Therefore, it is not possible to determine whether the observed changes in nutritional and bioimpedance parameters were directly attributable to EAA/KA supplementation or were influenced by other factors, including changes in dietary intake, concomitant treatments, or variations in patients’ clinical condition during follow-up.

The retrospective design represents a further limitation, as some potentially relevant variables, including C-reactive protein and interleukin-6, were not available. Inflammation may have acted as a confounding factor, particularly when interpreting changes in serum albumin and nutritional status. Ferritin was the only available indirect marker related to inflammation; however, its interpretation is complicated by its role as an iron-storage protein and its response to iron supplementation.

Another limitation of this study was the lack of a detailed assessment of dietary intake, such as a three-day dietary record, which would have allowed a more accurate quantification of patients’ energy and protein intake. Nevertheless, the MIS incorporates qualitative assessments of dietary intake and gastrointestinal symptoms in items 2 and 3, respectively. These components provide information on changes in food intake and appetite, as well as on the presence of gastrointestinal symptoms such as nausea and vomiting. Although this approach does not allow a precise quantification of actual dietary intake, changes in these MIS components suggested improvements in appetite and a reduction in gastrointestinal symptoms in some patients during the observation period. These findings may help contextualize the observed improvement in overall nutritional status, although they cannot establish an increase in energy or protein intake (Appendix A, Table A2).

The main findings after six months of treatment were a significant increase in serum albumin levels (*p* < 0.001; mean difference, +0.39 g/dL) and a significant reduction in the MIS (*p* = 0.003; mean difference, −5.50). These findings suggest a possible improvement in nutritional status during EAA/KA supplementation.

An increase in serum albumin levels was also reported by Sook et al. [21] in HD patients with hypoalbuminemia (serum albumin < 3.8 g/dL) treated with EAA/KA, with a mean increase of 0.2 g/dL. Similarly, Zakar et al. [23], in a cohort of 42 HD patients receiving EAA/KA at a dose of 0.1 g/kg body weight/day, reported a significant improvement in serum albumin levels among patients with baseline values below 35 g/L. PTH levels also showed a trend towards improvement during supplementation, while nutritional status improved, as indicated by better SGAscores.

The increase in serum albumin observed in our study may be clinically relevant. In addition to its role as a nutritional marker, albumin contributes substantially to plasma oncotic pressure and therefore plays a role in maintaining intravascular fluid balance. Improved albumin levels could potentially support plasma refilling during HD; however, this mechanism cannot be established from the present data. The observed reductions in ECW, both in absolute terms and as a percentage of total body water (ECW%), are consistent with a change in hydration status, although they do not directly demonstrate improved vascular refilling or hemodynamic stability.

Body weight and total body water (TBW) remained essentially unchanged over the observation period, with small, non-significant decreases. In conjunction with the reduction in ECW and ECW%, these findings may indicate a change in fluid distribution rather than a substantial reduction in total body water. The significant decrease in the Na/K and the ECM/BCM provides additional information on changes in body composition and hydration status. Nevertheless, these impedance-derived parameters should be interpreted cautiously, as they do not independently establish a redistribution of water into the intracellular compartment or an improvement in muscle quality.

Regarding calcium–phosphate metabolism, serum calcium levels increased significantly but remained within the normal range. As EAA/KA formulations are administered as calcium salts, their calcium content should be considered when interpreting this finding. Ketoanalogues may also be relevant to phosphate management in patients with CKD, but the increase in serum phosphorus observed at T6, although not statistically significant, cannot be attributed specifically to increased dietary phosphate intake without additional dietary data. Calcium, phosphorus, and PTH should therefore be monitored during supplementation, particularly in patients receiving other treatments that affect mineral metabolism.

Other nutritional interventions have also shown potential benefits in HD patients with PEW. Renal-specific oral nutritional supplements (RS-ONS) have been associated with increased serum albumin levels and reduced MIS [37]. In-center administration of oral nutritional supplements during HD has also been associated with lower hospital readmission rates among patients with severe hypoalbuminemia following hospital discharge [38]. Furthermore, intradialytic administration has been reported to be well tolerated, despite concerns about potential hypotension [39]. Although RS-ONS were used in our dialysis center to support malnourished patients until a few years ago, they are currently no longer available at our center. Consequently, patients who may benefit from this nutritional intervention may need to purchase these products independently, which could limit access for those unable to afford the associated costs.

Intradialytic parenteral nutrition (IDPN), when combined with oral nutritional supplementation, has been associated with improvements in prealbumin, serum albumin, body weight, spontaneous oral intake, and MIS, as well as with favorable clinical outcomes in some studies [40,41,42]. However, IDPN is generally intended to complement, rather than replace, oral nutritional intake. Its administration requires consideration of the additional fluid volume, which may be particularly relevant in patients with hypertension or fluid overload. Blood glucose monitoring is also important in patients with diabetes.

Overall, nutritional management and exercise interventions based on the concept of rehabilitation nutrition are important components of the management of malnutrition in HD patients [43,44]. Oral or intravenous protein supplementation may also help address inadequate nutrient intake and interrupt the cycle of PEW [45,46]. Within this context, our findings suggest that EAA/KA supplementation may be associated with improvements in selected nutritional and bioimpedance parameters in elderly patients undergoing maintenance HD. However, given the retrospective design, small sample size, absence of a control group, and potential confounding factors, these findings should be considered preliminary and require confirmation in larger prospective controlled studies.

## 5. Conclusions

In this small retrospective observational study of patients undergoing maintenance HD, six months of EAA/KA supplementation was associated with a significant increase in serum albumin levels and significant reductions in the MIS and the ECM/BCM. A significant decrease in ECW% was also observed, suggesting a possible improvement in hydration status.

These findings suggest that EAA/KA supplementation may be a promising nutritional strategy for patients undergoing maintenance hemodialysis who are at nutritional risk. However, given the small sample size, the presence of substantial comorbidities, the retrospective design, and the absence of a control group, these results should be interpreted with caution. Further prospective controlled studies involving larger patient populations are needed to confirm these preliminary findings and clarify the potential role of EAA/KA supplementation in the nutritional management of patients undergoing maintenance hemodialysis.

## Figures and Tables

**Figure 1:**
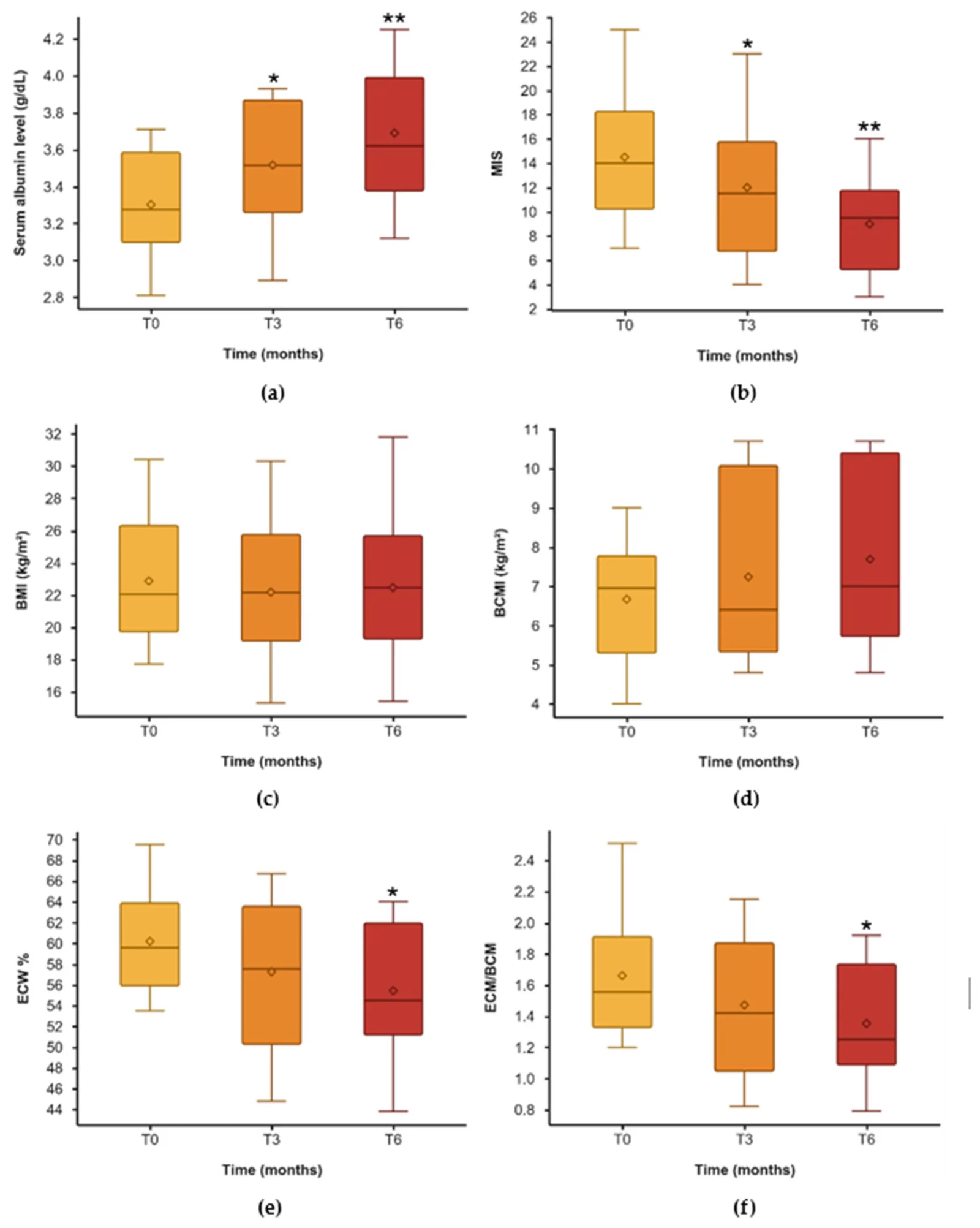
Longitudinal changes in nutritional status, anthropometric parameters and body composition from baseline (T0) to 3 months (T3) and 6 months (T6). (**a**) Serum albumin level, (**b**) MIS, (**c**) BMI, (**d**) BCMI, (**e**) ECW%, (**f**) ECM/BCM. Box plots represent the distribution of values, while dots indicate mean values. Statistical significance of changes over time is reported as *p*-values. * *p* < 0.05; ** *p* < 0.001.

**Table 3:** Comparison of nutritional, anthropometric, dialysis, biochemical and body composition parameters between baseline (T0) and 6 months (T6).

	Normal Values (*)	T0 (n.10) Mean ± SD	*95% CI*	T6 (n.10) Mean ± SD	*95% CI*	*p*
**Malnutrition assessment tool**						
MIS	<7	14.5 ± 5.6	*10.5–18.5*	9 ± 4.2	*6–12*	***0.003***
**Anthropometry**						
Body Weight (kg)		60.8 ± 14	*50.8–70.9*	59.9 ± 15.8	*48.6–71.2*	*0.390*
BMI (kg/m^2^)	18–25	22.87 ± 4	*20–25.7*	22.5 ± 4	*19.2–25.8*	*0.318*
**Dialysis**						
Kt/V sp	>1.2	1.5 ± 0.3	*1.3–1.7*	1.5 ± 0.2	*1.4–1.7*	*0.948*
nPCR sp (g/kg/die)	>1.2	1 ± 0.3	*0.8–1.2*	1.1 ± 0.3	*0.9–1.3*	*0.479*
Serum Urea preHD (mg/dL)	10–50	123.5 ± 29	*102.7–144.3*	138.1 ± 49.1	*102.9–173.2*	*0.345*
**Biochemistry**						
Serum Albumin (g/dL)	3.9–4.6	3.3 ± 0.3	*3.1–3.5*	3.7 ± 0.4	*3.4–3.9*	***<0.001***
Hemoglobin preHD (g/dL)	12–16	10.3 ± 1	*9.6–11*	10.9 ± 1.2	*10–11.8*	*0.276*
Lymphocytes (#/μL)	1–4.8 × 10^3^	1340 ± 829	*747–1933*	1350 ± 735	*824–1876*	*0.943*
Transferrin (mg/dL)	202–306	170.8 ± 42.7	*140.2–201.3*	170.1 ± 34.3	*145.6–194.6*	*0.911*
Ferritin (ng/mL)	10–291	512.5 ± 480.1	*169.2–855.9*	348.5 ± 298.1	*135.2–561.8*	*0.156*
Total Cholesterol (mg/dL)	<190	124.1 ± 26.9	*104.8–143.4*	131.1 ± 29.7	*109.8–152.3*	*0.084*
Triglycerides (mg/dL)	<150	86.9 ± 24.7	*69.2–104.6*	108.7 ± 44.9	*76.6–140.8*	*0.135*
Calcium (mg/dL)	8.1–10.4	8.7 ± 0.5	*8.3–9.1*	9.1 ± 0.5	*8.7–9.5*	***0.024***
Phosphorus (mg/dL)	2.7–4.5	3.9 ± 0.9	*3.3–4.6*	4.2 ± 1.5	*3.1–5.2*	*0.688*
PTH (pg/mL)	14.9–56.9	153.4 ± 64.7	*107.1–199.7*	128.4 ± 67.3	*80.3–176.5*	*0.364*
**Impedance analysis**						
FFM (kg)	(**)	45.8 ± 10	*38.7–53*	46.5 ± 11	*38.6–54.4*	*0.624*
TBW (L)	(**)	36.4 ± 8.7	*30.2–42.7*	34.6 ± 8.1	*28.8–40.4*	0.223
ECW (L)	(**)	21.7 ± 4.4	*18.6–24.9*	18.9 ± 3.5	*16.4–21.4*	***0.024***
ECW%	(**)	60.2 ± 5.4	*56.3–64.1*	55.4 ± 6.4	*50.9–60*	***0.025***
BCM (kg)	(**)	17.9 ± 5.9	*13.7–22.1*	20.6 ± 7.6	*15.2–26.1*	*0.070*
FM (kg)	(**)	15 ± 8.7	*8.7–21.2*	13.4 ± 6.9	*8.4–18.3*	*0.266*
PA (°)	4.5–6.3	3.7 ± 0.7	*3.2–4.1*	4.3 ± 1	*3.6–5*	***0.029***
Na/K	0.9–1	1.7 ± 0.3	*1.5–1.9*	1.4 ± 0.3	*1.2–1.6*	***0.025***
BCMI (kg/m^2^)	(**)	6.7 ± 1.6	*5.5–7.8*	7.7 ± 2.2	*6.1–9.3*	*0.078*
MM (kg)	(**)	22.9 ± 8.5	*16.8–29*	21 ± 7.4	*15.7–26.3*	*0.211*
ASMM (kg)	(**)	17 ± 5.7	*12.9–21.1*	16.4 ± 5.1	*12.7–20*	*0.405*
ECM/BCM	0.9–1.2	1.7 ± 0.4	*1.3–2*	1.3 ± 0.4	*1.1–1.6*	***0.034***

(*) Values in HD patients before the first dialysis session of the week. (**) Normal values are calculated based on the patient’s sex, height, age, and level of physical activity. Bold type indicates section headings and statistically significant *p*-values (*p* < 0.05). Italics indicate 95% confidence intervals (CIs) and *p*-values.

**Table 4:** Mean differences and 95% confidence intervals for clinical, biochemical and body composition parameters from T0 to T6.

	Mean Difference T6-T0	95% CI	
		LL	UL
MIS	−5.50	**−8.56**	**−2.44**
Body Weight (kg)	−0.94	−3.29	1.41
BMI (kg/m^2^)	−0.41	−1.29	0.47
Kt/V sp	−0.01	−0.20	0.21
nPCR sp (g/kg/die)	0.08	−0.17	0.33
Serum Urea preHD (mg/dL)	14.6	−18.6	47.8
Serum Albumin (g/dL)	0.39	**0.24**	**0.53**
Hemoglobin preHD (g/dL)	0.59	−0.56	1.74
Lymphocytes (#)	10	−300	320
Transferrin (mg/dL)	−0.7	−14.5	13.1
Ferritin (ng/mL)	−164	−404	75.7
Total Cholesterol (mg/dL)	7	−1.15	15.1
Triglycerides (mg/dL)	21.8	−8.24	51.8
Calcium (mg/dL)	0.37	**0.06**	**0.68**
Phosphorus (mg/dL)	0.24	−1.07	1.55
PTH (pg/mL)	−25	−84.1	34.1
FFM (kg)	0.68	−2.80	4.16
TBW (L)	−1.84	−5.02	1.34
ECW (L)	−2.83	**−5.19**	**−0.47**
ECW%	−4.76	**−8.78**	**−0.74**
BCM (kg)	2.71	−0.27	5.69
FM (kg)	−1.62	−4.71	1.47
PA (°)	0.67	**0.08**	**1.26**
Na/K	−0.28	**−0.52**	**−0.04**
BCMI (kg/m^2^)	1.02	−0.14	2.18
MM (kg)	1.91	−1.3	5.11
ASMM (kg)	0.64	−1.01	2.29
ECM/BCM	−0.31	**−0.59**	**−0.03**

## Data Availability

Data is unavailable due to privacy.

## Abbreviations

The following abbreviations are used in this manuscript:

ASMM	Appendicular Skeletal Muscle Mass
BCM	Body Cell Mass
BCMI	Body Cell Mass Index
BMI	Body Mass Index
CCI	Charlson Comorbidity Index
CKD	Chronic Kidney Disease
EAA-KA	Essential Amino Acids and Keto-Analogues
ECW	Extracellular Water
ECW%	Extracellular Water percentage of Total Body Water
EPO-α	Erythropoietin-α
ESKD	End-Stage Kidney Disease
FCM	Ferric Carboxymaltose
FFM	Fat-Free Mass
FM	Fat Mass
HD	Hemodialysis
IDPN	Intradialytic Parenteral Nutrition
KDIGO	Kidney Disease Improving Global Outcome
Kt/V sp	Dialytic clearance single pool
MIS	Malnutrition–Inflammation Score
MM	Muscle Mass
Na/K	Sodiumto–Potassium Ratio
nPCR sp	Normalized Protein Catabolic Rate single pool
ONS	Oral Nutrition Supplements
PA	Phase Angle
PEW	Protein Energy Wasting
PTH	Parathormone
RRF	Residual Renal Function
Rz	Resistance
SFO	Sucroferric oxyhydroxide
SGA	Subjective Global Assessment
TBW	Total Body Water
TIBC	Total Iron-Binding Capacity
Xc	Reactance

## Appendix A. Management of HD Patients—Clinical Practice and Nutritional Considerations

HD is generally initiated in patients with end-stage kidney disease (ESKD) when kidney function is no longer sufficient to maintain adequate metabolic, fluid, and electrolyte homeostasis. Upon initiating HD at our Dialysis Centre, patients undergo a nutritional assessment by a nutritionist, who prescribes an individualized dietary plan. According to the KDOQI 2020 Clinical Practice Guidelines, the recommended targets for metabolically stable adults undergoing maintenance HD are a protein intake of 1.0–1.2 g/kg body weight/day and an energy intake of 25–35 kcal/kg body weight/day. In ourcenter, dietary plans are generally designed to provide 1.2 g protein/kg body weight/day and 30 kcal/kg body weight/day [11].

Dietary phosphorus intake is managed according to patients’ serum phosphorus levels and individual nutritional requirements. A daily phosphorus intake of approximately 800 mg may be recommended when clinically appropriate. In addition to high-biological-value protein sources, plant-based proteins are encouraged where suitable, as phosphorus from plant foods generally has lower bioavailability than phosphorus from animal-derived foods and phosphate additives.

Nutritional assessment includes laboratory investigations, BIA, and completion of the MIS form (Table A1). This assessment is performed at the initiation of HD and subsequently every three months.

**Laboratory parameters.** Blood samples are collected before the first HD session following the longest interdialytic interval. Pre- and post-dialysis serumurea measurements obtained during this session are used to calculate single-pool Kt/V (spKt/V), an index of dialysis adequacy, and the normalized protein catabolic rate (nPCR), an indirect estimate of protein intake under conditions of metabolic stability, using the single-pool equations described by Daugirdas.

The timing of blood sampling also allows assessment of biochemical changes occurring over the interdialytic interval, including potentially elevated serum potassium and phosphorus levels before dialysis. These measurements support the nephrologist in evaluating electrolyte control and tailoring dietary advice and dialysis management to the individual patient.

**Bioelectrical impedance analysis (BIA).** BIA is performed approximately 20 min after the end of the midweek hemodialysis session, using a standardized post-dialysis time interval to improve the consistency of measurements. This timing was chosen because excessive fluid accumulation during the long interdialytic interval may prevent patients from reaching their prescribed dry weight by the end of the subsequent dialysis session. Performing BIA after the midweek session allows assessment of patients’ hydration and nutritional status under conditions in which the prescribed dry weight is more likely to have been achieved, thereby reducing the potential influence of interdialytic fluid overload on the measurements. Standardizing the time between the end of dialysis and BIA is also important because post-dialysis fluid redistribution may affect bioelectrical impedance measurements.

**Table A1:** MIS questionnaire.

Comprehensive Malnutrition Inflammation Score				
Points	0	1	2	3
A. Patients’ related medical history:				
**1. Change in end dialysis dry weight (overall changes in past 3–6 months)**	No decrease in dry weight or weight loss <0.5 kg	Minor weight loss (>0.5 kg but <1kg)	Weight loss more than 1 kg but <5%	Weight loss >5%
**2. Dietary intake**	Good appetite and no deterioration of the dietary intake pattern	Somewhat sub-optimal solid diet intake	Moderate overall decrease to full liquid diet	Hypo-caloric liquid to starvation
**3. Gastrointestinal (GI) symptoms**	No symptoms with good appetite	Mild symptoms, poor appetite or nauseated occasionally	Occasional vomiting or moderate GI symptoms	Frequent diarrhea or vomiting or severe anorexia
**4. Functional capacity (nutritionally related functional impairment)**	Normal to improved functional capacity, feeling fine	Occasional difficulty with baseline ambulation, or feeling tired frequently	Difficulty with otherwise independent activities (e.g., going to bathroom)	Bed/chair-ridden, or little to no physical activity
**5. Co-morbidity including number of years on dialysis**	On dialysis less than 1 year and healthy otherwise	Dialyzed for 1–4 years, or mild co-morbidity (excluding MCC *)	Dialyzed > 4 years, or moderate co-morbidity (including 1 MCC *)	Any severe, multiple co-morbidity (2 or more MCC *)
**B. Physical Exam (according to SGA criteria)**				
**6. Decreased fat stores or loss of subcutaneous fat (below eyes, triceps, biceps, chest)**	Normal (no change)	Mild	Moderate	Severe
**7. Signs of muscle wasting (temple, clavicle, scapula, ribs, quadriceps, knee, interosseous)**	Normal (no change)	Mild	Moderate	Severe
**C. Body Mass Index**				
**8. BMI**	BMI ≥ 20 kg/m^2^	BMI: 18–19.99 kg/m^2^	BMI: 16–17.99	BMI < 16
**D. Laboratory parameters**				
**9. Serum Albumin**	Serum Albumin: ≥4.0 g/dL	Serum Albumin: 3.5–3.9 g/dL	Serum Albumin: 3.0–3.4 g/dL	Serum Albumin: <3.0 g/dL
**10. TIBC or serum transferrin**	TIBC ≥ 250 mg/dL or serum transferrin ≥ 200	TIBC: 200–249 mg/dL or serum transferrin 170–199 mg/dL	TIBC: 150–199 mg/dL or serum transferrin 140–169 mg/dL	TIBC < 150 mg/dL or serum transferrin < 140 mg/dL

* MCC (Major Comorbid Conditions) include Chronic Heart Failure class III or IV, full blown AIDS, severe Coronary Artery Disease, moderate to severe Chronic Obstructive Pulmonary Disease, major neurologic sequelae, and metastatic malignancies or s/p recent chemotherapy. Bold type is used to distinguish the different sections of the MIS and its 10 individual items.

The analysis of individual MIS components showed statistically significant reductions in scores for changes in post-dialysis dry weight, dietary intake, gastrointestinal symptoms, signs of muscle wasting, and serum albumin after six months of follow-up. No statistically significant changes were observed in functional capacity, comorbidity, fat stores, BMI, or total iron-binding capacity/serum transferrin. The results for all individual MIS components are presented in Table A2.

**Table A2:** Changes in individual MIS components between baseline (T0) and six months (T6).

	T0	T6	
MIS Component	Median (IQR)	Median (IQR)	*p*-Value (Wilcoxon)
**1. Change in end dialysis dry weight**	2 (1.750)	0 (0.750)	0.017
**2. Dietary intake**	1 (0.675)	1 (1.000)	0.042
**3. Gastrointestinal symptoms**	1 (1.000)	0 (0.000)	0.015
**4. Functional capacity**	1 (0.000)	1 (1.000)	0.588
**5. Co-morbidity including number of years on dialysis**	2 (1.750)	2 (1.750)	0.773
**6. Decreased fat stores or loss of subcutaneous fat**	2 (2.500)	1 (2.000)	0.120
**7. Signs of muscle wasting**	1.5 (1.000)	1 (0.750)	0.037
**8. BMI**	0 (0.750)	0 (1.000)	1.00
**9. Serum Albumin**	2 (0.750)	1 (0.750)	0.006
**10. TIBC or serum transferrin**	1.5 (1.750)	2 (1.750)	0.773

Bold type is used to distinguish the different sections of the MIS and its 10 individual items.

**Table A3:** Contents of one EAA/KA tablet and intake per kgBW/day at the administered dose.

	Contents of 1 Tablet (mg)	Intake Per kgBW/Day (mg)
D,L-alpha-ketoisoleucine, calcium salt (mg)	67	6.7
Alpha-ketoleucine, calcium salt (mg)	101	10.1
Alpha-ketophenylalanine, calcium salt (mg)	68	6.8
Alpha-ketovaline, calcium salt (mg)	86	8.6
D,L-Alpha-hydroxymethionine, calcium salt (mg)	59	5.9
L-lysine (in the form of L-lysine acetate) (mg)	75	7.5
L-threonine (mg)	53	5.3
L-tryptophan (mg)	23	2.3
L-histidine (mg)	38	3.8
L-tyrosine (mg)	30	3
**Total**	600	60
Nitrogen (mg)	36	3.6
Calcium (mg)	50	5
